# Comparison of AI software tools for automated detection, quantification and categorization of pulmonary nodules in the HANSE LCS trial

**DOI:** 10.1038/s41598-024-78568-z

**Published:** 2024-11-13

**Authors:** Rimma Kondrashova, Filip Klimeš, Till Frederik Kaireit, Katharina May, Jörg Barkhausen, Susanne Stiebeler, Jonathan Sperl, Sabine Dettmer, Frank Wacker, Jens Vogel-Claussen

**Affiliations:** 1 Institute for Diagnostic and Interventional Radiology (OE 8220), Hannover Medical School, Carl-Neuberg-Str. 1, 30625 Hannover, Germany; 2 German Centre for Lung Research (DZL), Biomedical Research in End-stage and Obstructive Lung Disease Hannover (BREATH), Hannover, Germany; 3 Department of Radiology and Nuclear Medicine, University Hospital Schleswig-Holstein, Lübeck, Germany; 4 Department of Thoracic Oncology, Hospital Grosshansdorf, Grosshansdorf, Germany; 5 MR Application Predevelopment, Siemens Healthcare GmbH, Erlangen, Germany

**Keywords:** Lung cancer, Population screening

## Abstract

Participant management in a lung cancer screening (LCS) depends on the assigned Lung Imaging Reporting and Data System (Lung-RADS) category, which is based on reliable detection and measurement of pulmonary nodules. The aim of this study was to compare the agreement of two AI-based software tools for detection, quantification and categorization of pulmonary nodules in an LCS program in Northern Germany (HANSE-trial). 946 low-dose baseline CT-examinations were analyzed by two AI software tools regarding lung nodule detection, quantification and categorization and compared to the final radiologist read. The relationship between detected nodule volumes by both software tools was assessed by Pearson correlation (*r*) and tested for significance using Wilcoxon signed-rank test. The consistency of Lung-RADS classifications between Software tool 1 (S1, Aview v2.5, Coreline Soft, Seoul, Korea) and Software tool 2 (S2, Prototype ‘’ChestCTExplore’’, software version ToDo, Siemens Healthineers, Forchheim, Germany) was evaluated by Cohen’s kappa (*κ*) and percentual agreement (*PA*).The derived volumes of true positive nodules were strongly correlated (*r* > 0.95), the volume derived by S2 was significantly higher than by S1 (*P* < 0.0001, mean difference: 6mm^3^). Moderate *PA* (62%) between S1 and S2 was found in the assignment of Lung-RADS classification (*κ* = 0.45). The *PA* of Lung-RADS classification to final read was 75% and 55% for S1 and S2, but the incorporation of S1 into the initial nodule detection and segmentation must be considered here. Significant nodule volume differences between AI software tools lead to different Lung-RADS scores in 38% of cases, which may result in altered participant management. Therefore, high performance and agreement of accredited AI software tools are necessary for a future national LCS program.

**Supplementary Information:**

The online version contains supplementary material available at 10.1038/s41598-024-78568-z.

## Introduction

Lung cancer is the leading cause of cancer death worldwide^1,2^.

If lung cancer is detected at an early stage, it is more likely to be treated curatively. According to the American Thoracic Society, screening for lung cancer is recommended for people between 50 and 80 years old and in fairly good health with at least a 20-pack-year smoking history who currently smoke or have quit in the past 15 years^1^. During the last years, multiple large multicenter lung cancer screening (LCS) studies worldwide such as The National Lung Screening Trial in the US, Dutch-Belgian Randomized Lung Cancer Screening Trial (NELSON), Italian Lung Cancer Screening Trial (ITALUNG) or German Lung Cancer Screening Intervention Trial (LUSI) concluded that lung cancer screening using low-dose computed tomography (LDCT) reduces lung cancer mortality^2–6^. Recently a holistic northern German interdisciplinary lung cancer screening study (HANSE)^7^ has been started for optimal definition of the high-risk screening population and implementation of the screening workflow in the current national healthcare infrastructure.

Lung nodule management in the HANSE study depends on the Lung Imaging Reporting and Data System (Lung-RADS 1.1) assessment^7–9^. Five categories to discriminate between high-risk and low-risk nodules are part of the Lung-RADS assessment. After the detection of a nodule and correct recognition of nodule type, an accurate measurement of the size of detected nodules is a prerequisite for subsequent successful nodule management^10^. Recent studies have shown that classification based on mean nodule diameter leads to a massive overestimation of true nodule size. In contrast, nodule volumetry demonstrates lower inter-observer variability compared to manual bidimensional diameter measurements. Therefore classification based on volume is recommended^11,12^. Horeweg et al.‘s research established the pivotal role of the 100 mm³ threshold as the cornerstone for various guidelines including NELSON, British Thoracic Society (BTS) and Lung-RADS guidelines^13^. As our study follows the Lung-RADS 1.1 standard, we adopted for thresholds of 113 mm³ and 34 mm³, which are extrapolated from diameter measurements.

Manual 3D volume segmentation of pulmonary nodules is time-consuming and error-prone; therefore, radiologists are nowadays assisted by artificial intelligence (AI)-based software tools when reading LDCT examinations^14–17^. There are currently 17 products on the market that can detect, quantify, and categorize pulmonary nodules with distinct detection performance^18^. Accurate detection and quantification are essential for the correct categorization of detected nodules. If a different Lung-RADS grade is assigned to the same subject by different software, the participant may be treated differently. In some cases, variability in the volumetric assessment of pulmonary nodules may result in a false-positive or false-negative diagnosis^19^. Such inconsistency may occur, for example, when different software is used among different centers as parts of a national screening program^20^. With the wide range of AI software available, it is important to know if there are significant differences in lung nodule detection, quantification and classification between the different AI tools.

The purpose of this study was to investigate and compare the performance of two AI-based software tools regarding lung nodule detection, quantification, and categorization in the HANSE trial population prior to the implementation of a national lung cancer screening program in Germany^21^.

## Method

### Study population

The study participants were part of the HANSE LCS trial^22^, a northern Germany multicenter LCS study. In this study, 946 LDCT randomly selected baseline participant examinations, performed (between July 2021 and July 2022) in a German Cancer Society-certified lung cancer center at Hannover Medical School, were retrospectively analyzed. The institutional review board of all participating institutions approved the HANSE study and all study participants provided written informed consent. The methods employed in this study conform to the principles outlined in the Declaration of Helsinki. The image acquisition details are listed in the Supplement.

### LDCT evaluation

After the first reading of LDCTs by one experienced radiologist with specialized training in LCS, a computer-aided detection (CAD) technology of Software tool 1 (S1, Aview v2.5, Coreline Soft, Seoul, Korea) automatically detected, segmented, and classified pulmonary nodules. The nodule candidates suggested by CAD technology, were flagged by the radiologist as either correct- or false-positive findings. Subsequently, the false positives were excluded from the report. Further, nodules were automatically classified into solid, non-solid, calcified and part-solid groups according to their attenuation. Inaccuracies in the measurement of volume as well as the nodule group affiliation were corrected using a threshold-based algorithm if necessary. Based on the measured volume and assigned nodule group, an individual nodule Lung-RADS score was determined. The overall Lung-RADS score for each participant was defined by the highest individual nodule Lung-RADS score. Cases classified as 3 or 4 according to Lung-RADS 1.1 scoring system^23^ were reviewed by another experienced board-certified thoracic radiologist; therefore, this was not an independent, blinded second read. In case of nodule classification discrepancy between both readers, the case was presented to the radiological conference for a consensus evaluation. The final decision, consisting of the first read by the radiologist, AI-assistance and the second read of Lung-RADS 3–4 cases, was considered as a final reading (FR) and defined the reference standard for comparison of AI software tools. The reference standard corresponds to that of the HANSE trial.

Similarly to S1, software tool 2 (S2, Prototype ‘’ChestCTExplore’’, software version ToDo, Siemens Healthineers, Forchheim, Germany) was used for automated nodule detection, segmentation and classification. Both software tools were trained using deep learning and were dedicated for oncology, incidental pulmonary nodules and LCS^24^. While S1 was used prospectively, S2 analyzed the results of the LDCT retrospectively using the same criteria. A difference between the two software tools for determining volumes lies in the position of the start and end points: S1 measures from voxel center to voxel center, whereas S2 measures from the outer edge of the voxels. S1 uses the voxel count and S2 also applies a partial volume correction. The nodule type determination methods differ between S1 and S2. S1 employs a classification-based method using the random-forest algorithm^25^, while S2 utilizes a segmentation-based approach. In S2, two segmentation masks are generated: the first includes total nodules, and the second includes solid-core nodules. Nodule types are determined in S2 based on logical operations between these two masks.

True Positive (TP) nodules were identified as those detected in the FR dataset. Nodules detected by the S1 / S2 but not confirmed by the FR dataset were defined as false positives (FP). Nodules found in the FR dataset but not in the S1 / S2 dataset were designated as false negatives (FN).

The matching of detected nodules from the S1 / S2 dataset to the FR dataset is described in the Supplement. No manual correction was made for the matching of data outputs.

### Statistical analysis

Statistical analysis was performed with JMP Pro 16 software (SAS Institute, Cary, NC).

### Nodule detection

Nodule detection performance was assessed by sensitivity and positive predictive value (PPV) between FR and S1, FR and S2 of all detected nodules.

An identical sub-analysis was performed for clinically relevant nodules between FR and S1 and FR and S2. Filters of 34mm^3^ and 113mm^3^ volume were applied. These thresholds correspond to the Lung-RADS 1.1 scoring system.

Additionally, in the group of nodules ≥ 34mm^3^ volume, the same analysis was executed for nodule subgroups (solid, non-solid, calcified and part-solid).

### Nodule quantification

After excluding the FP nodules, both software tools (S1 and S2) and the FR dataset were compared regarding nodule volume. In the S1 vs. S2 comparison, only nodules detected by both software tools were included. Nodule volume parameters were tested for normality using the Shapiro-Wilk test. Since both parameters were not normally distributed, a non-parametric paired Wilcoxon signed-rank test was used to assess volume median differences in the following comparisons: FR vs. S1, FR vs. S2 and S1 vs. S2. *P* values < 0.05 were considered as a statistically significant difference. Further, the mean differences were quantified by Bland-Altman analysis and the association between the measured volumes was explored by Pearson correlation analysis (*r*). The relative volumetric errors between the final reading and the two software tools were computed as a percentage difference between two measuring volumes divided by the mean of the two values.

The same analysis was also conducted for TP nodules after the application of 34mm^3^ and 113mm^3^ volume thresholds.

### Nodule categorization

On the participant and individual nodule level, the agreement of Lung-RADS classification of both software systems (S1, S2) with the ground truth (FR) and between each other (S1 vs. S2) was determined by Cohen’s kappa coefficient (κ) and percentual agreement (PA). PA was defined as a fraction of the number of Lung-RADS classifications in agreement and the total number of Lung-RADS classifications. Finally, the fraction was converted to PA by multiplication with 100. At the nodule level, when comparing the two software tools with each other, only the correctly positive nodules detected by both software tools were scored for fair comparison.

Consistently, the identical analysis was conducted at the nodule level for nodules with volume ≥ 34mm^3^ and ≥ 113mm^3^.

Additionally, on the participant level, the whole analysis was repeated for participants with a total Lung-RADS score ≥ 3 and participants with a total Lung-RADS score < 3.

Further details regarding the statistics of Lung-RADS misclassification at the individual nodule level and false-positive rate on the participant level are presented in the Supplement.

## Results

To reflect the real world of LCS both nodules and masses (tumors with diameter larger than three cm) were examined. The volume of the smallest detected nodule and the largest mass was 0.3mm^3^ and 18.9cm^3^ respectively. In the study population of 946 participants, a total of 3345 lung nodules were found in the FR dataset and 765 out of 946 subjects had at least one nodule. The number of detected nodules ranged from 1 to 86 per subject. Of the 3345 nodules, 1174 were found clinically relevant (nodule volume ≥ 34mm^3^) based on the FR. Besides 791 solid nodules, 258, 112, and 13 were non-solid, calcified, and part-solid, respectively. Of 1174 clinically relevant nodules, 282 nodules had volumes greater than 113mm^3^. Five masses were found in the final read of which three were detected by each software tool.

### Nodule detection

**Fig. 1:**
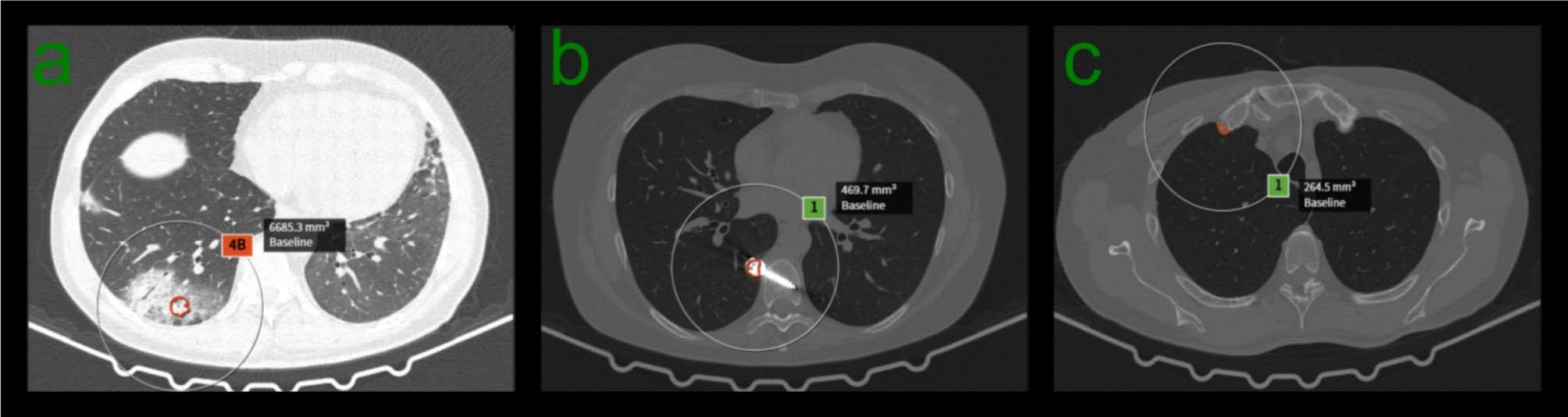
Examples of FP detection for both software tools. In (**a**) a consolidation due to pneumonia, in (**b**) a metallic foreign body and in (**c**) an osteophyte cases were detected by both software tools as a pulmonary nodule.

**Fig. 2:**
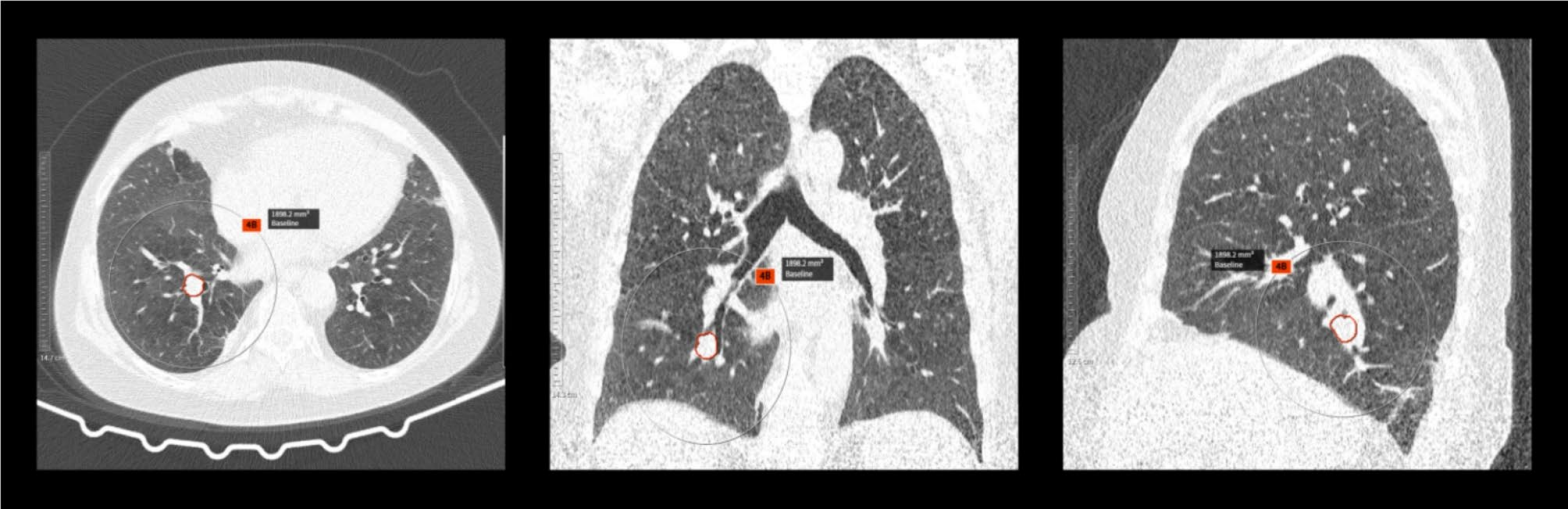
Exemplary FN detection of pulmonary solid nodule (volume 1898.2 mm^3^, Lung-RADS 4B category) for both software tools for a 67-year-old female patient. No malignancy was found in the biopsy.

**Fig. 3:**
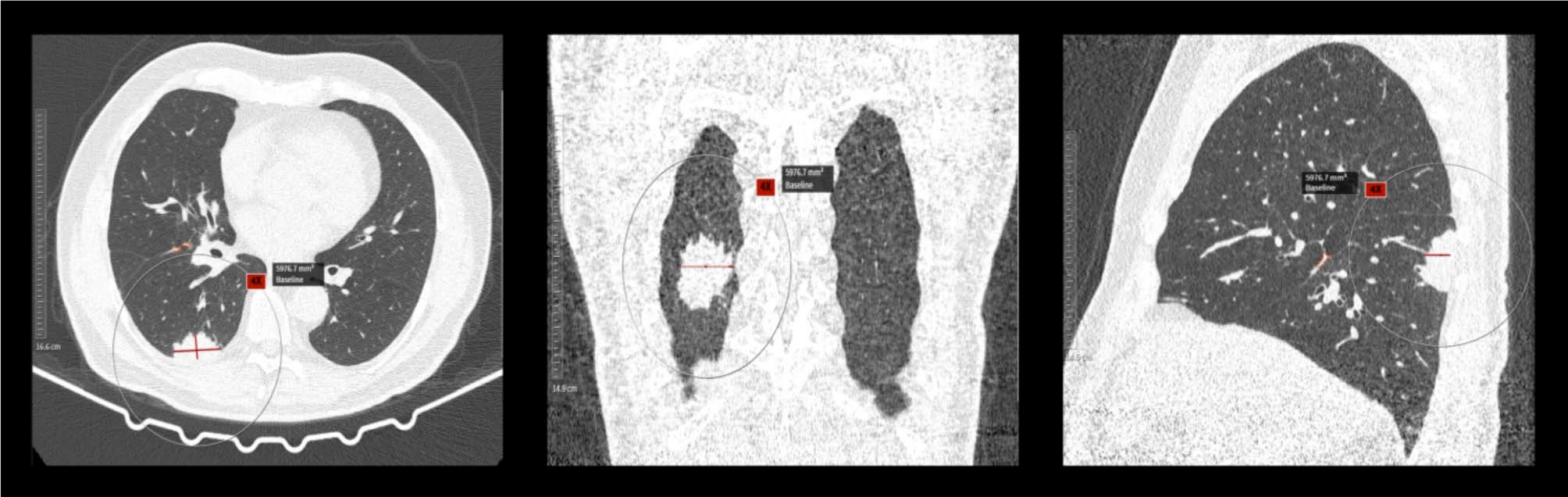
Exemplary FN detection of pulmonary solid mass (volume 5976.7 mm^3^, Lung-RADS 4X category) for S1 software tool for a 69-year-old patient. After CT-assisted transthoracic puncture, the histology showed adenocarcinoma.

Examples of FP and FN pulmonary nodule detection are shown in Figs. 1, 2 and 3. Sensitivities and PPVs of the S1 software tool were higher when compared to the S2 software tool for all nodules, for nodules with volume ≥ 34mm^3^ and nodules with volume ≥ 113mm^3^ (see Table 1). Table 2 shows detection results for clinically relevant nodules ≥ 34mm^3^ concerning their type. Sensitivities and PPVs were higher for S1 for all nodule types. The lowest sensitivities were observed for part-solid and non-solid using S1 and S2, respectively. For both software tools, the highest PPV was found for non-solid nodules.

**Table 1:** Detection performance of both software tools in all nodules, in nodules with volume ≥ 34 mm^3^ and nodules with volume ≥ 113 mm^3^.

Software tool	Nodule volume	TP	FP	FN	Sensitivity [%]	PPV [%]
S1	All	2142	316	1203	64	87
	≥ 34 mm^3^	1032	210	142	88	83
	≥ 113 mm^3^	234	105	48	83	69
S2	All	1526	538	1819	46	74
	≥ 34 mm^3^	782	459	392	66	63
	≥ 113 mm^3^	202	305	80	72	40

FN, false negative; FP, false positive; PPV, positive predictive value; S1, software tool 1 dataset; S2, software tool 2 dataset; TP, true positive.

**Table 2:** Detection performance of both software in subgroups of clinically relevant nodules with volume ≥ 34 mm^3^.

Software tool	Nodule type	TP	FP	FN	Sensitivity [%]	PPV [%]
S1	Solid	720	175	71	91	80
	Non-Solid	207	16	51	80	92
	Calcified	97	18	15	87	84
	Part-Solid	8	1	5	62	80
S2	Solid	570	321	221	72	63
	Non-Solid	121	9	137	47	93
	Calcified	83	57	29	74	59
	Part-Solid	8	3	5	61	72

FN, false negative; FP, false positive; PPV, positive predictive value; S1, software tool 1 dataset; S2, software tool 2 dataset; TP, true positive.

### Nodule quantification

**Fig. 4:**
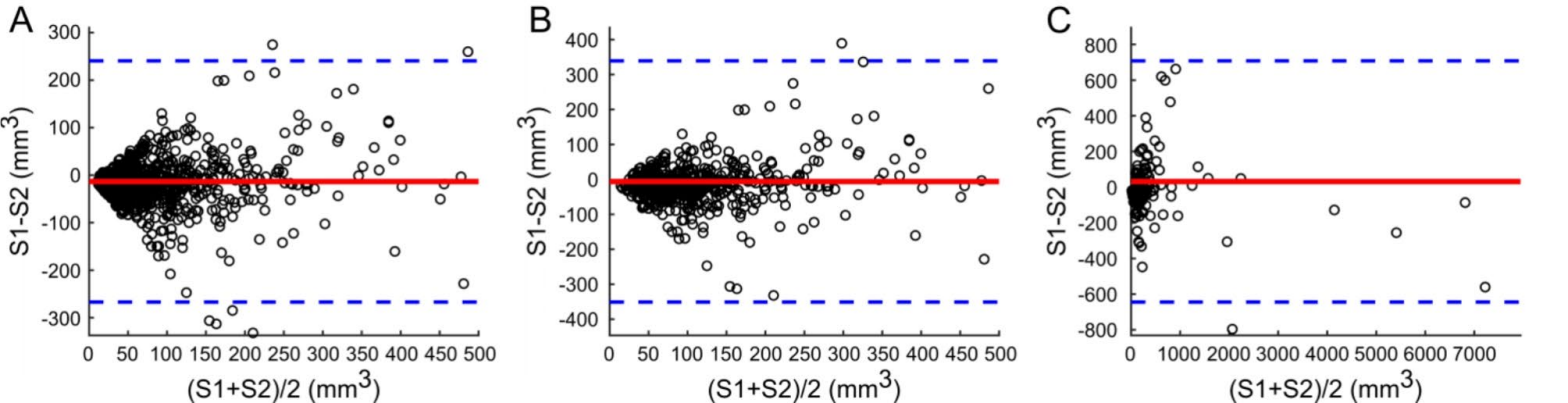
Bland-Altman analysis of volume for all nodules (**A**), for nodules with volume ≥ 34 mm^3^ (**B**) and nodules with volume ≥ 113 mm^3^ (**C**) between software tools S1 and S2. The full red line indicates the mean difference (-13.3 (**A**), -6.0 (**B**) and 31.6 (**C**) mm^3^) and blue dotted lines indicate 95% limits of agreement.

Although the derived volumes of all TP nodules from S1 and S2 datasets were strongly correlated (all *r* > 0.95), the volume derived by S2 was significantly larger than that obtained by S1 (both *P* < 0.0001, see Table 3), except for the nodules with volume ≥ 113mm^3^. In Fig. 4, Bland-Altman analysis between S1 and S2 software tools is depicted.

**Table 3:** Comparison of detected TP nodules regarding median and mean volume for FR, S1 and S2 datasets.

		Median (IQR)	Mean (SD)	Mean Bias (relative difference in %)	*P* value^a^	Pearson correlation *r*
All	S1	35.6 (52.6)	97.7 (418.4)	-13.3 (-47%)	< 0.0001*	0.95
	S2	58.8 (52.8)	111.1 (375.8)			
≥ 34 mm^3^	S1	63.0 (73.0)	164.4 (566.9)	-6.0 (-25%)	< 0.0001*	0.95
	S2	85.3 (73.9)	170.5 (509.2)			
≥ 113 mm^3^	S1	183.6 (204.9)	470.6 (1079.5)	31.6 (-10%)	0.31	0.95
	S2	199.5 (159.2)	439.0 (971.8)			
All	FR	35.3 (51.9)	106.9 (446.9)	-15.7 (-46%)	< 0.0001*	0.81
	S2	56.0 (52.6)	122.5 (580.6)			
≥ 34 mm^3^	FR	68.9 (72.0)	191.2 (612.6)	-7.6 (-15%)	< 0.0001*	0.81
	S2	86.1 (77.0)	198.8 (803.6)			
≥ 113 mm^3^	FR	198.6 (211.6)	565.1 (1125.9)	29.7 (11%)	0.14	0.80
	S2	209.2 (177.1)	535.5 (1533.2)			
All	FR	32.4 (47.8)	88.5 (527.3)	9.5 (4%)	0.24	0.59
	S1	31.5 (48.5)	79.0 (343.4)			
≥ 34 mm^3^	FR	67.1 (63.6)	164.8 (752.3)	22.6 (10%)	0.0037*	0.58
	S1	61.7 (69.7)	142.3 (486.6)			
≥ 113 mm^3^	FR	186.2 (168.3)	517.6 (1530.1)	106.4 (21%)	0.0083*	0.55
	S1	177.4 (172.1)	411.2 (973.1)			

FR, final reading dataset; IQR, interquartile range; Mean bias, mean difference derived from Bland-Altman analysis; S1, software tool 1 dataset; S2, software tool 2 dataset; SD, standard deviation; *r*, Pearson correlation coefficient.

^a^Paired Wilcoxon signed rank test, significantly different measurements (*P* < 0.05) are marked with *. It should be noted that each comparison exclusively incorporates TP nodules detected either by both software tools or by software tool and final read. Consequently, this leads to changed volume numbers for the same software tool across various comparisons.

**Fig. 5:**
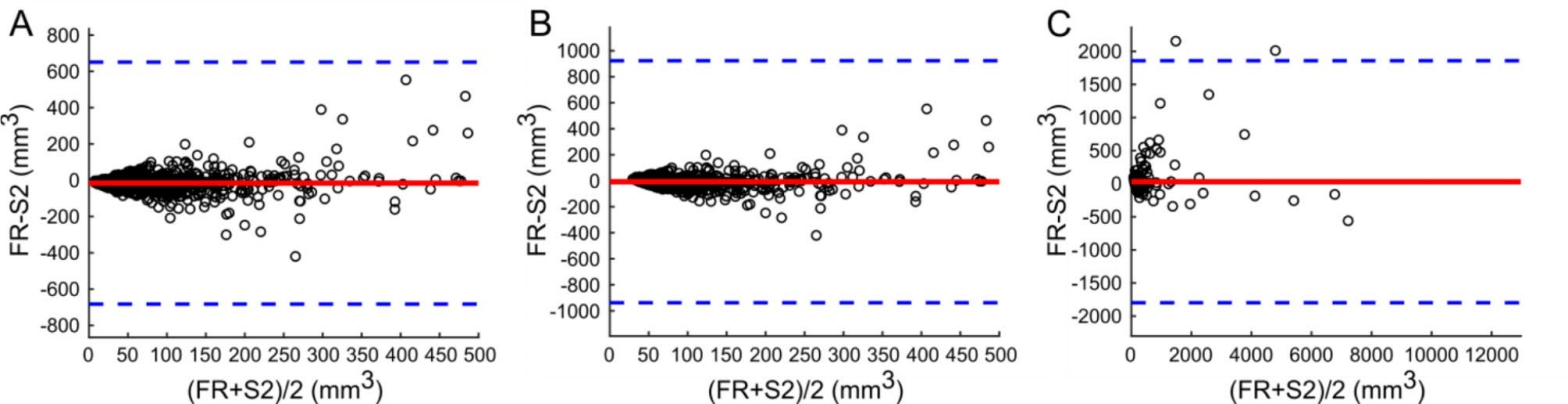
Bland-Altman analysis of volume for all nodules (**A**), for nodules with volume ≥ 34 mm^3^ (**B**) and for nodules with volume ≥ 113 mm^3^ (**C**) between FR and software tool S2. The full red line indicates the mean difference (-15.7 (**A**), -7.6 (**B**) and 29.7 (**C**) mm^3^) and blue dotted lines indicate 95% limits of agreement.

In comparison between FR and S2 datasets (see Fig. 5), all volumes obtained by the S2 software tool were significantly larger (both *P* < 0.0001, Table 3), except for nodules with volume ≥ 113mm^3^.

**Fig. 6:**
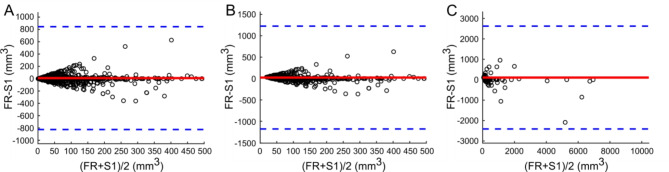
Bland-Altman analysis of volume for all nodules (**A**), for nodules with volume ≥ 34 mm^3^ (**B**) and for nodules with volume ≥ 113 mm^3^ (**C**) between FR and software tool S1. The full red line indicates the mean difference (9.5 (**A**), 22.6 (**B**) and 106.4 (**C**) mm^3^) and blue dotted lines indicate 95% limits of agreement.

Comparing the S1 dataset with the FR dataset (Fig. 6), FR volume measurements were found significantly higher (both *P* < 0.0037, Table 3), except for the comparison of all nodules.

### Nodule categorization

Supporting Information Table S1 shows the individual nodule categorization comparison according to its size, measured by volume. There was a good agreement in comparison of S1 and S2 datasets (all PA > 67%, all κ > 0.61). Higher Lung-RADS consensus was achieved between FR vs. S1 (all PA > 69%, κ > 0.58) when compared to FR vs. S2 (all PA > 55%, κ > 0.44). For all comparisons, the Lung-RADS agreement was decreased with nodule size.

Similarly, on a participant level, a moderate agreement of Lung-RADS categorization was observed in the comparison of S1 and S2 datasets (all PA > 54%, all κ > 0.41, see Table 4). As shown in Table 4, the comparison of FR vs. S1 reached a slightly higher agreement (all PA > 60%, all κ > 0.40) than the comparison of FR vs. S2 (all PA > 55%, all κ > 0.28).

**Table 4:** Comparison of Lung-RADS categorization on a patient level.

Comparison	Lung-RADS selection	Cohen’s κ	PA [%]
S1 vs. S2	Lung-RADS ≥ 3	0.45	54
	Lung-RADS < 3	0.41	64
	All	0.45	62
FR vs. S2	Lung-RADS ≥ 3	0.41	60
	Lung-RADS < 3	0.28	55
	All	0.33	55
FR vs. S1	Lung-RADS ≥ 3	0.40	60
	Lung-RADS < 3	0.53	76
	All	0.55	75

FR, final reading dataset; Lung-RADS, lung CT screening reporting & data system; PA, percent agreement; S1, software tool 1 dataset; S2, software tool 2 dataset.

For both software tools, the volume difference (*n* = 17 (65.4%) and *n* = 18 (69.2%) for software tool S1 and S2, respectively) was the most common reason for false nodule categorization with Lung-RADS score ≥ 3. Less frequent causes of classification differences were: false positive nodules (*n* = 5 (19.2%) and *n* = 6 (23.1%) for software tool S1 and S2, respectively), false nodule type classification (*n* = 3 (11.5%) and *n* = 0 (0%) software tool S1 and S2, respectively) and false negative nodules (*n* = 1 (3.8%) and *n* = 2 (7.7%) for software tool S1 and S2, respectively).

Incorrect categorization of nodules with LUNG-RADS score < 3 is presented in the Supplement.

## Discussion

The main findings of the present study include percentage disagreement in the assignment of Lung-RADS classification in 38% of participants as well as significant differences in volumetric measurement and sensitivity of pulmonary nodule detection when comparing S1 and S2.

There is currently a large body of data comparing computer-aided detection (CAD) in lung cancer screening with radiologist performance^24,26,27^, demonstrating that AI improves the lung nodule detection performance of radiologists. The study showed, that the nodule detection performance of S2 is less than that of S1, potentially impacting the reference read, if S2 was used instead of S1 in the HANSE-Study. Nonetheless, it is important to note that in this study the AI-aided read essentially functions as a form of assistance for the radiologist during the prolonged process of pulmonary nodule segmentation, and should not be considered a substitute for a human “reader”. In addition in this study, we found that sensitivity as well as PPV also differed when using the two examined software tools. Considering different nodule subgroups, both software tools showed a higher sensitivity concerning solid nodules than to non-solid nodules, whereas the sensitivity of clinically relevant part-solid^28^ nodules was similar by both software tools (61% and 62%). The decreased sensitivity in the part-solid group is likely to be influenced by the limited number of part-solid nodules found in the FR dataset, only 13 part-solid nodules were present in the whole cohort. Therefore, further studies are needed in the area of non-solid as well as part-solid nodules to increase detection performance in both nodule subgroups^29^.

Finally, false positive but also false negative rates represent a drawback of CAD algorithms, which may be crucial when choosing a software tool for an LCS program.

Successful patient management requires correct nodule measurement. In this study, the volume parameters were considered, as literature has demonstrated superior performance with lower inter-observer variability for volumetry compared to manual diametric measurements^12^. For TP nodules, S2 measured both parameters larger than S1, except for the volumes of the TP nodules with volume ≥ 113mm^3^, which were not statistically different. Identical results were observed for the comparison with FR with statistically higher volumes of the S2 software tool and significantly lower volumes for the S1 tool even for larger nodules (volume ≥ 113mm^3^). This supports the fact, that due to manual adjustment of the nodule size by the radiologist significant changes to volume were made. This is often necessary due to adjacent anatomic structures of the nodules such as pulmonary vessels for example. This finding clearly shows that there is still a radiologist interaction needed for visual inspection and quality control with the current performance of AI tools in the LCS setting.

In the study by Zhao et al.^19^, three software tools were compared with each other based on the median volume of the detected nodules on baseline scans in the LCS. The nodules were classified according to characteristics, such as location, attachment, shape and edge. Similarly to our results, the study showed significant differences in volumetry between software packages for nearly all nodule groups, except for non-smooth nodules. However, the clinical output regarding the Lung-RADS classification was not reported in detail. The volumetry differences in our study are also in agreement with the previously published results of Hoop et al.^30^. The authors evaluated six software packages for solid lung nodule volumetry and reported significant mean volume differences in 11 out of 15 possible pairs of software tools. Furthermore, Ashraf et al.^31^ demonstrated in their study that the reliability of volume measurements significantly declines when different algorithms are used, which is also congruent with our results.

The above-mentioned differences may lead to differences in Lung-RADS classification and thus potentially to different clinical treatments of the participants. In the study of van Riel et al.^23^ interobserver disagreement in the Lung-RADS category, however without using artificial intelligence, was seen in one-third of the examined scan pairs and led to different patient management in 8%. Therefore, when using different AI software tools in a national screening program, high agreement of all accredited AI software tools is mandatory.

In our study, the differences in total Lung-RADS categorization between the two software tools were clinically relevant. Both software tools assigned the same category to the same participant in just 62% of the nodules. Our finding is in agreement with a recent phantom study by Peters et al.^32^, which found, that participant management may be influenced by an incorrectness of a CAD System. The authors showed the inconsistency in assigning a Lung-RADS score between two software tools in 14.9%, which is lower than our results, likely due to a different study design.

Comparing the individual software tool regarding the clinically relevant score (Lung-RADS ≥ 3) in each case with the FR - the value agrees with both software tools in 60%. That means in 40% of the cases the radiologist changed the score. For Lung-RADS levels ≥ 3, S1 nodule size measurement values were significantly lower and S2 significantly higher compared to the final result of the radiologist. In both cases, the incorrect measurement of the volume by one of the software tools was the reason in about 65%. Looking at the Lung-RADS stages 1 and 2, in more than 90% of the cases the errors were due to detection (false positive and false negative nodules) issues and in the wrong subgroup classification (especially calcified, non-solid subgroups). Only in the remaining 10% of cases, volume measurement differences caused incorrect Lung-RADS classification.

At the individual nodule level, the differences were less severe. When comparing both software tools, there was a difference of 33% for the nodules with volume > 113mm^3^ and 23% for the nodules with volume > 34mm^3^. Comparing both software tools with FR, especially nodules with volume > 11mm^3^ were incorrectly assigned to Lung-RADS scores 3 and 4a.

If Lung-RADS score 3 entails a 6-month LDCT for control, 4a entails a control in 3 months or, if malignancy is highly probable, a PET CT. For category 4b or 4x, a tissue biopsy is performed if the probability of malignancy is high, or a PET CT or chest CT with or without contrast is performed if the probability of malignancy is low^33^. With such a high inconsistency of 46% in the Lung-RADS classification from Lung-RADS Score 3 onwards (comparison S1 vs. S2 on participant level), participants may be treated differently at different centers using different software tools. In a recent publication, Hwang et al. analyzed an actual nationwide LCS situation in South Korea. They concluded: there is a high inter-institutional variability in the interpretation of LCS results partially explained by different usage of the same CAD-system (e.g., disagreement in the Lung-RADS category occurred in 50.6% of the participants)^20^. If the Lung-RADS classification is falsely high, this may lead to unnecessary psychological strain on the participant, unnecessary radiation exposure and even invasive measures as well as higher costs. If the classification is too low, this may lead to overlooking (potentially) malignant findings. The automated decision of the software tool regarding Lung-RADS categorization may also influence the opinion of the radiologist, resulting in clinically relevant consequences^34^.

This study had several limitations. Firstly, software tool S1 was used prospectively in the HANSE study itself and likely influenced the final decision (FR dataset) of the radiologist, which was used as a reference standard for comparisons. This fact could partly explain the bias in the comparison of the two software tools with FR. Undeniably, all nodule candidates detected by CAD systems, as well as volumes and Lung-RADS classification, are currently evaluated by the radiologist. However, AI may influence the radiologist’s opinion. Secondly, the distribution of nodule types regarding attenuation was in favor of solid nodules. A low number of part-solid nodules were included in the FR dataset. Therefore, the analysis in this subgroup might be examined in future studies. Also, only two available software tools were evaluated in this study. S1 was commercially available. Currently, there are more software packages available and they hold the potential to be subjects to future research.

## Conclusion

This study found that, despite the accurate detection of solid nodules, there were significant differences between AI software tools in measuring volume and as a consequence in assigning the Lung-RADS score. Significant nodule volume differences between AI software tools led to different Lung-RADS scores in 38% of cases, which may result in altered participant management. Our findings clearly show that there is still a radiologist interaction needed for visual inspection and quality control with the current performance of AI tools in the LCS setting. Therefore, high performance and agreement of accredited AI software tools and adequate user training are mandatory for successful integration in a future national LCS program.

## Electronic supplementary material

Below is the link to the electronic supplementary material.

Supplementary Material 1

## Data Availability

All data was acquired at Hannover Medical School, Germany, and is available in de-identified form from the corresponding author upon request. A formal data sharing agreement is needed. The MATLAB code is available from the corresponding author upon request. A formal code sharing agreement is needed.
