# Supplementary material for: Comparison of AI software tools for automated detection, quantification and categorization of pulmonary nodules in the HANSE LCS trial
Source: Sci Rep. 2024 Nov 13;14:27809. doi: 10.1038/s41598-024-78568-z (PMC11561337; doi:10.1038/s41598-024-78568-z)
Supplement: Supplementary file 1 — Supplementary Material 1 [file 41598_2024_78568_MOESM1_ESM.docx]

**Supporting Information**

**Image Acquisition**

Low-dose chest CT scans (100 kV, tin filtration, 300 mA, pitch 0.8, 0.33 s gantry rotation time) were performed using a mobile CT scanner (Siemens SOMATOM go.Top CT, Siemens Healthineers, Forchheim, Germany) serving three lung cancer centers in Hannover, Lübeck, and Grosshansdorf. Scans were acquired during a 3-second breath-hold in inspiration. The LDCT images were reconstructed at 1 mm slice thickness using sinogram affirmed iterative reconstruction (SAFIRE2) with the lung kernel (Br64).

**Patient and nodule characterization**

The study cohort consisted of 946 randomly selected CT scans. All our participants (mean age: 64 years, age range: 55-79, 57.6% males) met the criteria of the Hanse study, which are available in the supplementary material of the Ref. 9. 56 % of all participants were active smokers. Mean smoking history of all participants was 41 years (± 8 years) with mean cigarettes per day of 22 (± 9). Regarding the noduli localization: 709 noduli were in lower left lobe, 743 noduli were in upper left lobe, 745 noduli in lower right lobe, 313 in middle right lobe and 835 in upper right lobe.

Nodule matching

For nodule detection comparison, an automated in-house-developed program in MATLAB (MATLAB 2020b, Mathworks, Natick, Massachusetts, USA) was used. For each detected nodule of S1 / S2 dataset, nodule centroid coordinates and volume were extracted and compared to same measures of possible paired nodule from FR / S1 dataset. The nodule with highest volume overlap was considered as a matched pair.

False-positive rate calculation and its results

On the patient level, false-positive rate was calculated as the ratio of false-positive classified participants to the sum of false-positive and true-negative classified participants. True-negative participants were defined as participants without noduli and correctly identified by the software tool. False-positive classified participants were defined as participants without noduli and false identified by the software tool. The false-positive rate was 11.6% and 34.2% for software tool S1 and S2, respectively.

Nodule categorization details on possible Lung-RADS misclassification and its statistics

Lung-RADS category 4X involves additional morphological attributes, that increase suspicion of malignancy. Such characteristics should be visually evaluated by a radiologist, therefore 4B and 4X categories were considered as one category.

Considering the Lung-RADS classification at the individual nodule level, the following factors can lead to the change of the total Lung-RADS score for each patient:

- The nodule turned out to be a false positive or was missed by the software tool

- The volume of the nodule was measured larger or smaller

- The classification into the subgroups (solid, part-solid, non-solid or calcified nodules) was incorrect

All of these factors were quantified and evaluated for both software tools.

Nodule diameter quantification results

Supporting Information Table S1 shows comparison of detected TP nodules based on diameter for FR, S1 and S2 datasets. The mean diameter obtained by S2 was significantly higher than that measured by S1 (all *P* < 0.0001, Supporting Information Figure S1), however the derived mean diameters of S1 and S2 correlated well (all *r* >0.85).

In the comparison between FR and S2 datasets (Supporting Information Figure S2), all diameters obtained by S2 software tool were significantly elevated (all *P* < 0.0001).

Comparing S1 dataset with FR dataset (Supporting Information Figure S3), FR mean diameter measurements were found significantly higher (all P < 0.01).

Incorrect nodule categorization with Lung-RADS score < 3

For nodules with Lung-RADS score < 3 false positive detection was the most common reason for incorrect nodule categorization of both software tools (n=73 (34.0%) and n=186 (47.0%) for software tools S1 and S2, respectively). Other incorrect nodule categorization cases were associated with false negative detection (n= 62 (28.8%) and n=90 (22.7%) for software tool S1 and S2, respectively) and false nodule type (n=60 (27.9%) and n=85 (21.5%) for software tool S1 and S2, respectively). The volume difference was the least frequent cause of classification inconsistencies for both software tools (n= 20 (9.3%) and n=35 (8.8%) for software tool S1 and S2, respectively).

**Supporting Information Tables**

**Supporting Information Table S1.**  Comparison of Lung-RADS categorization on individual nodule level.

| Comparison | Nodule volume | Cohen’s κ | PA [%] |
| --- | --- | --- | --- |
| **S1 vs S2** | All | 0.63 | 83 |
|  | ≥ 34 mm^3^ | 0.61 | 77 |
|  | ≥ 113 mm^3^ | 0.63 | 67 |
| **FR vs S2** | All | 0.57 | 81 |
|  | ≥ 34 mm^3^ | 0.46 | 73 |
|  | ≥ 113 mm^3^ | 0.44 | 55 |
| **FR vs S1** | All | 0.80 | 93 |
|  | ≥ 34 mm^3^ | 0.68 | 88 |
|  | ≥ 113 mm^3^ | 0.58 | 69 |
| FR, final reading dataset; PA, percent agreement; S1, software 1 dataset; S2, software 2 dataset. | | | |
